# Older Age, Polypharmacy, and Low Systolic Blood Pressure Are Associated With More Hypotension-Related Adverse Events in Patients With Type 2 Diabetes Treated With Antihypertensives

**DOI:** 10.3389/fphar.2021.728911

**Published:** 2021-09-24

**Authors:** Martina Ambrož, Sieta T. de Vries, Klaas Hoogenberg, Petra Denig

**Affiliations:** ^1^ Department of Clinical Pharmacy and Pharmacology, University of Groningen, University Medical Center Groningen, Groningen, Netherlands; ^2^ Department of Internal Medicine, Martini Hospital, Groningen, Netherlands

**Keywords:** type 2 diabetes, systolic blood pressure, adverse events, elderly, sex differences, polypharmacy, overtreatment

## Abstract

**Background and Aims:** Low systolic blood pressure (SBP) levels while being treated with antihypertensives may cause hypotension-related adverse events (hrAEs), especially in the elderly, women, and frail patients. We aimed to assess the association between the occurrence of hrAEs and low SBP levels, age, sex, and polypharmacy among patients with type 2 diabetes (T2D) treated with antihypertensives.

**Methods:** In this cohort study, we used the Groningen Initiative to ANalyse Type 2 diabetes Treatment (GIANTT) database which includes patients managed for T2D in primary care from the north of the Netherlands. Patients treated with ≥1 antihypertensive drug and ≥1 SBP measurement between 2012 and 2014 were included. The outcome was the presence of an hrAE, i.e. postural hypotension, dizziness, weakness/tiredness, and syncope in 90 days before or after the lowest recorded SBP level. Age (≥70 vs. <70 years), sex (women vs. men), polypharmacy (5–9 drugs or ≥10 drugs vs. <5 drugs), and SBP level (<130 or ≥130 mmHg) were included as determinants. Logistic regression analyses were conducted for age, sex and polypharmacy, including the SBP level and their interaction, adjusted for confounders. Odds ratios (OR) with 95% confidence intervals (CI) are presented.

**Results:** We included 21,119 patients, 49% of which were ≥70 years old, 52% were women, 57% had polypharmacy, 61% had an SBP level <130 mmHg and 5.4% experienced an hrAE. Patients with an SBP level <130 mmHg had a significantly higher occurrence of hrAEs than patients with a higher SBP level (6.2 vs. 4.0%; ORs 1.41, 95%CI 1.14–1.75, 1.43, 95%CI 1.17–1.76 and 1.33, 95%CI 1.06–1.67 by age, sex, and polypharmacy, respectively). Older patients (OR 1.29, 95%CI 1.02–1.64) and patients with polypharmacy (OR 5–9 drugs 1.27, 95%CI 1.00–1.62; OR ≥10 drugs 2.37, 95% CI 1.67–3.37) were more likely to experience an hrAE. The association with sex and the interactions between the determinants and SBP level were not significant.

**Conclusion:** Low SBP levels in patients with T2D treated with antihypertensives is associated with an increase in hrAEs. Older patients and those with polypharmacy are particularly at risk of hrAEs. Age, sex, and polypharmacy did not modify the risk of hrAEs associated with a low SBP level.

## Introduction

Blood pressure targets for patients with type 2 diabetes (T2D) are commonly lower in comparison to the general population because of their increased risk of cardiovascular (CV) morbidity and mortality (Cryer et al., 2016; American Diabetes Association, 2020; Cosentino et al., 2020). Several guidelines and clinical trials suggest to lower SBP below 130 or even 120 mmHg in all patients with T2D, implying that the benefits outweigh possible risks of treatment (Beckett et al., 2011; Warwick et al., 2015; Xie et al., 2016; Bangalore et al., 2017; Williams et al., 2018; American Diabetes Association, 2020). However, there are concerns that treatment to low SBP levels increases the occurrence of adverse events (AEs) (Benetos et al., 2016; Sinclair et al., 2019; Group et al., 2021). A meta-analysis from 2016 which included almost 180,000 participants, several of which had T2D, observed that a reduction of SBP below 130 mmHg prevents one major CV event but is associated with six treatment discontinuations due to intercurrent conditions or serious AEs (Thomopoulos et al., 2016). Further, lower SBP levels have been associated with higher mortality in T2D patients older than 75 years vs. 60–75 years treated with antihypertensive drugs (van Hateren et al., 2010), which suggests that the optimal SBP target may differ across subpopulations. Also, the occurrence of treatment-related AEs seems to differ between patient groups since studies have shown a higher risk of drug-related AEs among women (Fan et al., 2008; Zopf et al., 2008; Yu et al., 2016; de Vries et al., 2019), older, and frail patients (Zopf et al., 2008; Davies and O'Mahony, 2015; Holm et al., 2017; Sink et al., 2018).

Several studies from clinical practice show that up to 20% of patients with T2D have SBP levels <130 mmHg while receiving multiple antihypertensive drugs or medication treatment intensification (Kerr et al., 2012; de Vries et al., 2014; Sonmez et al., 2020). This percentage is even higher in the elderly or frail, where more than half of the patients have SBP levels <130 mmHg (Sussman et al., 2015; McCracken et al., 2017). These low SBP levels can lead to hypotension-related AEs, including syncope, tiredness, and postural hypotension (Group et al., 2015; Bangalore et al., 2017), and could indicate overtreatment with antihypertensives. Although one might expect that specific patient groups are more vulnerable for these AEs when they are treated to low blood pressure levels, no significant age-by-treatment interaction effect was seen in adults included in the SPRINT trial (Sink et al., 2018). However, participants with diabetes, history of stroke, heart failure, dementia or standing SBP less than 110 mmHg were excluded from this trial. Since T2D can affect the CV and renal system, patients with T2D may have a different risk of AEs from antihypertensive treatment than those without T2D (Wu et al., 2009). Whether the occurrence of hypotension-related AEs in T2D patients treated to low SBP levels is affected by age or other patient characteristics is unknown.

Our aim was to assess the association between the occurrence of hypotension-related AEs and low SBP levels, age, sex, and polypharmacy among patients with T2D treated with antihypertensives in general practice. Our first hypothesis was that patients with low SBP levels but also older patients, women, and those with polypharmacy more often experience a hypotension-related AE. Furthermore, we aimed to assess whether age, sex, and polypharmacy influence the association between low SBP levels and hypotension-related AEs. We hypothesized that the risk of hypotension-related AEs when having low SBP levels is intensified in older patients, females, and those with polypharmacy. Insight in possible differences in such risks among patient groups is important to guide more personalized treatment of hypertension in patients with T2D.

## Materials and Methods

### Study Design and Population

In this cross-sectional cohort study, we used the Groningen Initiative to ANalyse Type-2 diabetes Treatment (GIANTT; www.giantt.nl) database. This database contains anonymous electronic medical records data of patients managed for T2D in primary care from the northern part of the Netherlands.

We included patients with at least one SBP measurement between the years 2012 and 2014. The day of the lowest SBP measurement in this time period was defined as index date. In case the lowest SBP level was recorded multiple times, the date of the first measurement was used. Patients had to have a practitioner confirmed diagnosis of T2D before the index date, had to be 18 years or older at the index date, and had to have at least 90 days of medical history before and 90 days of follow-up after index date to be included in our study. Patients without a prescription of an antihypertensive drug (anatomic therapeutic chemical (ATC) classification codes C02, C03, C07, C08, C09) in 90 days before the index date were excluded. Data were available from 189 general practices in the study period, after excluding data from three practices that had not documented any hypotension-related diagnostic codes in the study period.

We obtained an exemption letter for full ethical approval from the University Medical Center Groningen Medical Ethics Review Board (reference number M20.252895), since we used anonymous medical record data for this study.

### Outcome Variable

Our primary outcome was the presence of a hypotension-related AE in the 90 days before or after index date. This time window was chosen because an AE may be documented after the measurement of a low SBP, or the blood pressure may have been measured after the occurrence of an AE. The AEs were chosen based on the literature (Group et al., 2015; Bangalore et al., 2017), and defined with International Classification of Primary Care (ICPC) diagnostic codes used in Dutch primary care. The following diagnostic codes were included as hypotension-related AEs: K88 (postural hypotension), N17 (dizziness, vertigo), A04 (weakness, tiredness, lethargy), and A06 (syncope).

### Determinants

Age (≥70 vs. <70 years), sex (women vs. men), polypharmacy (polypharmacy (5–10 drugs) or hyper polypharmacy (≥10 drugs) vs. no polypharmacy) and SBP level (<130 mmHg vs. ≥130 mmHg) were included as determinants that may influence the occurrence of hypotension-related AEs. Age, sex and SBP level measured in the practice as documented at index date were used. Polypharmacy was based on the number of medications at the 3^rd^ pharmacological subgroup level of the ATC classification that a patient was prescribed in a period of 90 days up to the index date in addition to the one antihypertensive drug all patients had been prescribed by design.

### Confounders

The following patient characteristics available from the medical record data in GIANTT that may be associated with the selected AEs and with the SBP level and/or can differ between patients with different age, sex and polypharmacy, were included as potential confounders: glycated hemoglobin A1c (HbA1c) level (continuous variable), duration of diabetes (<10 years or ≥10 years), smoking status (smoker or non-smoker), diastolic blood pressure level (continuous variable), body mass index (BMI; continuous variable), presence of decreased estimated glomerular filtration rate (eGFR; ≤60 mL/min/1.73 m^2^; calculated using the serum creatinine from GIANTT and Chronic Kidney disease Epidemiology Collaboration formula or extracted from the database if creatinine levels were missing), presence of albuminuria (albumin creatinine ratio ≥30 mg/g or albumin in 24 h urine ≥300 mg), presence of dyslipidemia (defined as low density lipoproteins (LDL) ≥2.5 mmol/L), prescribed lipid lowering medication (none, 1 drug, ≥2 drugs) and glucose lowering medication (none, 1 oral drug, ≥2 oral drugs and/or insulin). Laboratory values were extracted as the last value in 180 days up to the index date or, in case that was not available, the first value in 90 days after index date. Diabetes duration was calculated on index date. Smoking status was assessed in the 180 days up to index date. BMI was calculated based on patients’ weight closest to the index date in the 5 years before or 1 year thereafter and the most recent height recorded any time before or after index date. If height and/or weight were not available, the BMI as entered in GIANTT was used. Presence of prescriptions was calculated in the 90 days up to index date.

### Missing Data

There were no missing values for the determinants and the primary outcome. Confounders with less than 30% of missing values were imputed using multiple imputation by chained equation (MICE) (White et al., 2011). Patients with a missing value for albuminuria (59%) were classified as not having albuminuria, since such testing is less likely in patients without expected kidney damage. None of the other confounders had more than 30% missing values.

### Analyses

Demographics were analyzed descriptively for patients with and without hypotension-related AEs. For each of the determinants, a logistic regression analysis was conducted including the SBP level and the interaction between SBP level and age, sex, and polypharmacy. These analyses were adjusted for the potential confounders to assess the odds ratios (ORs) for the occurrence of hypotension-related AEs. In the analysis of polypharmacy, there was no adjustment for glucose and lipid lowering therapy since these variables are part of the calculation of polypharmacy. In the analyses where age, sex, or polypharmacy were not used as a determinant, they were included as continuous (age and polypharmacy) or dichotomous (sex) confounding variables.

Several sensitivity analyses were conducted. First, we conducted a sensitivity analysis using a higher cut-off level for age of 80 years and using both higher and lower cut-off levels for SBP of 140 and 120 mmHg, respectively. Next, we expanded the definition of the outcome to include other less specific ICPC diagnostic codes that may be related to hypotension: A80 (trauma, injury), L75 (femur fracture), L76 (other fracture), L81 (musculoskeletal injury), S16 (bruise, concussion) and S17 (abrasion, scratch).

All analyses were conducted in Stata version 14 (Stata Corp., College Station, TX). *p*-values <0.05 were considered statistically significant and ORs with 95% confidence intervals (CIs) are presented.

## Results

We included 21,119 patients with T2D treated with antihypertensives who met our inclusion criteria (Supplementary Figure S1), of which 1,135 (5.4%) experienced a hypotension-related AE (Table 1). Forty nine percent of the included patients were older than 70 years, 52% were women, 57% had polypharmacy or hyper polypharmacy and 61% had the lowest SBP level below 130 mmHg. Patients who experienced a hypotension-related AE were more often women, older, had a longer diabetes duration and had more often eGFR ≤60 ml/min/1.73 m^2^ (Table 1). Almost half of the patients with a recorded AE had postural hypotension. Complete data were available for 52% of the patients.

**TABLE 1:** Patient characteristics.

	No adverse event (N = 19,984)	Adverse event (N = 1,135)
Female; N (%)	10,275 (51)	632 (56)
Lowest SBP in mmHg; mean ± SD	125 ± 14	121 ± 16
Lowest SBP <130 mmHg, N (%)	12,079 (60)	802 (71)
Age; mean ± SD	69 ± 11	71 ± 12
Age ≥70 years; N (%)	9,753 (49)	685 (60)
Polypharmacy; N (%)			
no	8,818 (44)	351 (31)
polypharmacy	9,277 (46)	574 (51)
hyper polypharmacy	1,889 (9)	210 (19)
Number of antihypertensives; N (%)
1	6,708 (34)	347 (31)
2	6,700 (34)	341 (30)
3 or more	6,576 (33)	447 (39)
HbA1c in %; mean ± SD	6.9 ± 1.0	7.0 ± 1.0
missing	976 (5)	62 (5)
Diabetes duration ≥10 years; N (%)	5,459 (27)	358 (32)
BMI in kg/m^2^; mean ± SD	30.4 ± 5.6	30.4 ± 5.5
missing	793 (4)	69 (6)
DBP in mmHg; mean ± SD	73 ± 10	71 ± 11
missing	216 (1)	10 (1)
eGFR ≤60 ml/min/1.73m^2^; N (%)	4,121 (21)	347 (31)
missing	4,045 (20)	143 (13)
Smoking; N (%)	2,797 (14)	150 (13)
missing	4,770 (24)	233 (21)
LDL cholesterol ≥2.5 mmol/L; N (%)	7,259 (36)	421 (37)
missing	5,718 (29)	298 (26)
Albuminuria; N (%)	396 (2)	21 (2)
missing	11,913 (60)	630 (56)
Hypotension related adverse event; N (%)			
Postural hypotension (K88)		534 (47)
Weakness, tiredness (A04)		336 (30)
Dizziness, vertigo (N17)		229 (20)
Syncope (A06)		117 (10)

SBP, systolic blood pressure; HbA1c, glycated hemoglobin A1c; BMI, body mass index; DBP, diastolic blood pressure; eGFR, estimated glomerular filtration rate; LDL, low-density lipoprotein.

### Associations With the Occurrence of Hypotension-Related AEs

**FIGURE 1:**
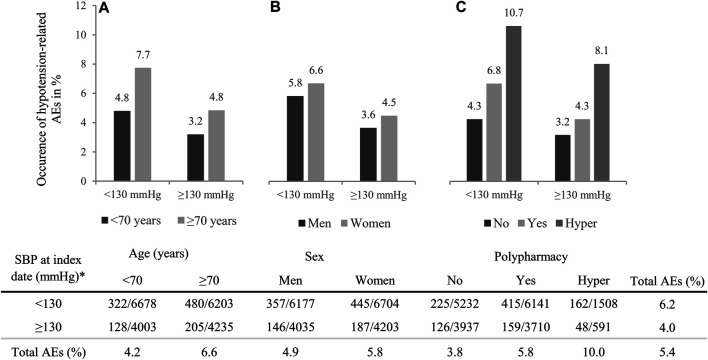
Occurrence of hypotension-related adverse events (AEs) per systolic blood pressure (SBP) level by **(A)** age, **(B)** sex and **(C)** polypharmacy. The table below presents the numbers of AEs per total number of patients in that group. *Index date is defined as the lowest SBP level between 2012 and 2014.

**FIGURE 2:**
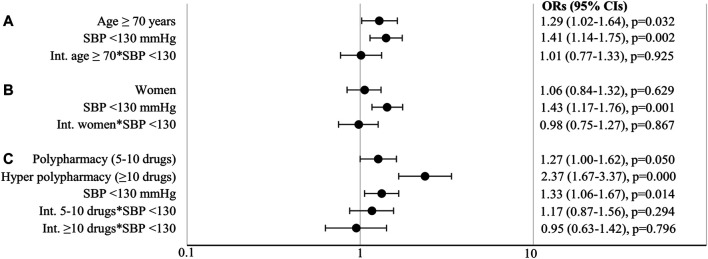
Odds ratios (OR) with 95% confidence intervals (CIs) and *p*-values for **(A)** age, **(B)** sex, **(C)** polypharmacy, with systolic blood pressure (SBP) and their interactions. Age and sex analyses were adjusted for glycated hemoglobin A1c, diabetes duration, body mass index, smoking, diastolic blood pressure, estimated glomerular filtration rate, glucose lowering therapy, dyslipidemia, lipid lowering therapy, albuminuria, number of comedication and sex or age; polypharmacy analysis was adjusted for the same variables except for glucose and lipid lowering therapy. Int., interaction.

Older patients more often experienced a hypotension-related AE than younger patients (6.6 vs. 4.2%; Figure 1A). In the logistic regression analysis, this main effect of age was statistically significant (OR 1.29, 95% CI 1.02–1.64; Figure 2A).

Women more often experienced a hypotension-related AE than men (5.8 vs. 4.9%; Figure 1B), but this difference was not statistically significant (OR 1.06, 95% CI 0.84–1.32; Figure 2B).

Patients prescribed more comedication more often experienced a hypotension-related AE (no polypharmacy 3.8%, polypharmacy 5.8% and hyper polypharmacy 10.0%; Figure 1C). In the logistic regression analyses, the effects of polypharmacy and hyper polypharmacy were statistically significant (OR polypharmacy vs. no polypharmacy 1.27, 95% CI 1.00–1.62 and OR hyper polypharmacy vs. no polypharmacy 2.37, 95% CI 1.67–3.37; Figure 2C).

Patients with SBP levels <130 mmHg more often experienced a hypotension-related AE than those with SBP ≥130 mmHg (6.2 vs. 4.0%; Figure 1). Statistically significant higher occurrence of AEs with lower SBP levels was shown in all conducted analyses (Figure 2): age (OR 1.41, 95% CI 1.14–1.75), sex (OR 1.43, 95% CI 1.17–1.76) and polypharmacy (OR 1.33, 95% CI 1.06–1.67).

### Modifying Effect of Age, Sex, and Polypharmacy on the Occurrence of AEs in Patient Treated to Low SBP Level

The interactions between the determinants and SBP level <130 mmHg were not statistically significant (OR for interaction with age 1.01, 95% CI 0.77–1.33 in Figure 2A; OR for interaction with sex 0.98, 95% CI 0.75–1.27 in Figure 2B; OR for interaction with polypharmacy 1.17, 95% CI 0.87–1.56 and OR for interaction with hyper polypharmacy 0.95, 95% CI 0.63–1.42 in Figure 2C). This indicates that older patients, women, and patients with polypharmacy or hyper polypharmacy are not at additional risk of hypotension-related AEs when having SBP levels <130 mmHg than younger patients, men, and patients with no polypharmacy when having low SBP levels.

### Sensitivity Analyses

The sensitivity analysis with a higher cut-off level for age showed that patients aged ≥80 years experienced more hypotension-related AEs than younger patients (7.8 vs. 4.8%), but this main effect was no longer statistically significant (OR 1.08, 95% CI 0.82–1.41; Supplementary Figures S2, S3).

The analysis using an SBP cut-off of 120 mmHg showed similar results as the main analysis (Supplementary Figures S4, S5). When using an SBP cut-off of 140 mmHg (Supplementary Figure S6) the effects of SBP level and age became non-significant (Supplementary Figure S7). Furthermore, patients with polypharmacy but not with hyper polypharmacy were at an additional risk of hypotension-related AEs at SPB levels <140 mmHg when compared to patients without polypharmacy (polypharmacy OR 1.52, 95% CI 1.02–2.28; hyper polypharmacy OR 1.16, 95% CI 0.66–2.04; Supplementary Figure S7). None of the other interactions were statistically significant.

Each of the additional AEs in the extended list occurred in 2–10% of patients who experienced an AE (Supplementary Table S1). The analyses including these additional AEs showed similar results as the main analyses (Supplementary Figures S8, S9).

## Discussion

This study among T2D patients treated with antihypertensives showed that older age, polypharmacy, and low SBP levels were all independently related to experiencing more hypotension-related AEs. The higher occurrence of hypotension-related AEs among patients with low SBP levels was not significantly aggravated by older age, female sex, or polypharmacy.

Several studies in non-diabetic populations have shown a higher occurrence of AEs in older patients (Zopf et al., 2008; Davies and O'Mahony, 2015; Holm et al., 2017) and in one study also no significant interaction between age and SBP level on AEs was observed (Sink et al., 2018). Our results showing a higher occurrence of AEs at older age without an interaction with SBP level are therefore in line with these previous studies. Nevertheless, a meta-analysis of clinical trials, several of which included T2D patients, showed an increased risk of hypotension in patients younger than 65 years, which they assumed was a consequence of more intensive antihypertensive treatment in younger patients (Thomopoulos et al., 2018). Although they observed slightly higher increment of discontinuations in the older patients, the ratio between risks and benefits was similar in older and younger patients. We found no significant differences in the occurrence of hypotension-related AEs between older and younger patients when using the SBP level 140 mmHg as a cut-off value. Our findings confirm the clinical trial data in a real-world setting of patients with T2D and suggests that lowering SBP levels below 140 mmHg seems safe in patients of all ages. Nevertheless, patients with T2D treated with antihypertensives reaching SBP levels below 130 mmHg should be closely monitored for the occurrence of hypotension-related AEs and possible overtreatment, regardless of age.

In our study, women had a slightly higher occurrence of hypotension-related AEs than men, but this difference was not significant after adjusting for possible confounders. This is not in line with other studies showing increased occurrence of AEs in women (Fan et al., 2008; Zopf et al., 2008; Holm et al., 2017; Sim et al., 2018; de Vries et al., 2019). Most of these studies, however, used different methods in reporting of AEs and often no adjustments were made for confounding of SBP level or age.

We saw a generally higher occurrence of hypotension-related AEs in patients prescribed more medication, which was independent of the SBP level. This is in line with several studies showing a higher occurrence of AEs in patients prescribed more medication or those with a greater comorbidity burden (Zopf et al., 2008; Sim et al., 2018; Sink et al., 2018; Hanlon et al., 2020). In one study, also no significant interaction between frailty and SBP levels on AEs was found (Sink et al., 2018). In itself, the occurrence of hypotension-related AEs in those prescribed more medication was high. Amongst those with hyper polypharmacy, almost 11% of patients with SBP level <130 mmHg and more than 8% of patients with SBP level ≥130 mmHg experienced a hypotension-related AE. Whether this is due to the actual large number of medication or underlying diseases in unknown, but it can cause a great burden on the healthcare system, the patients’ health state and their quality of life. Sufficient attention for negative effects of hypertension treatment in patients with hyper polypharmacy is warranted.

Overall, patients reaching low SBP levels had a higher occurrence of hypotension-related AEs then those with higher SPB levels. This is in line with previous studies and meta-analyses (ACCORD Study Group et al., 2010; Group et al., 2015; Bangalore et al., 2017; Frey et al., 2019). Of note is our finding that this was independent of the patients’ age, sex, and number of medications. This implies that attention for hypotension-related AEs is generally required in patients treated to low SBP levels. The occurrence of AEs is a common reason for poor medication adherence (Leporini et al., 2014). To increase the likelihood of adherence to the antihypertensive treatment, possible benefits and risks of treatment should be weighted, and a personalized SBP target should be discussed with the patient (American Diabetes Association, 2020) and occasionally reevaluated during treatment. Unless the patient is adequately informed about the benefits and possible AEs of intensive treatment and agrees with it, less intensive treatment with higher SBP targets should be considered.

The strength of our study is using real-world data from almost all T2D patients treated in a large number of general practices in the north of the Netherlands. It should be noted that this region consists mostly of Caucasian people. The results may not be generalizable to other populations. Further, we conducted several sensitivity analyses using different age and SBP level cut-offs and AE definitions to validate our findings and further explore the relationship between SBP and the occurrence of hypotension-related AEs. Several limitations mostly related to the use of a database with routinely recorded primary care data must be acknowledged. First, it is possible that the general practitioners were not aware of or did not record all AEs that were experienced by patients, or that there were errors in the coding. A comparison with a recent clinical trial (Sink et al., 2018) of patients without diabetes showed somewhat similar rates of hypotension (2.5% in our study compared to 1.6% in the clinical trial). For some AEs we observed lower occurrences, for example, syncope (0.6 vs. 1.8%, respectively). In general, we do not expect that the recording of AEs would differ across patients but some patients might report more AEs to their prescribers than others (Loikas et al., 2015). Also, although we selected AEs which are related to hypotension, we cannot guarantee that these AEs were caused by a low SBP level. We conducted a post hoc analysis using only those AEs which occurred at the same time or after the low SBP level was recorded to reduce the chance of the two events not being connected. This analysis revealed similar results (Supplementary Figures S10, S11). Nevertheless, there can be other causes for the AEs, also for the common postural hypotension in our study. Further, the number of SBP measurements varied between patients, with 2% of patients having only one measurement in the study period. It is not clear to what extent this might bias our findings. Next, some of the included confounders had almost a third of missing values. We used multiple imputation for these variables to reduce possible bias. Furthermore, we included polypharmacy as an indicator of comorbidity. Other measures, such as frailty, were unfortunately not recorded in our data. Last, we did not include the type of drug or drug dose or treatment duration in the analysis. Although this might explain part of the differences in the occurrence of AEs between different subpopulations, this is not expected to affect the associations between the SBP levels and hypotension-related AEs.

To conclude, the observed higher occurrence of hypotension-related AEs in older patients, patients with polypharmacy and those with low SBP levels indicates that there should be sufficient attention for hypotension-related AEs in those patients. Contrary to our expectation, age, sex, and polypharmacy did not increase the risk of hypotension-related AEs associated with a low SBP level in patients with type 2 diabetes. Possible negative effects of medication treatment to low SBP targets in clinical practice should be regularly evaluated in all patients with T2D. Personalized treatment targets may be warranted to reduce hypotension-related AEs, but also other underlying problems and treatment options should be explored with these patients.

## Data Availability

The datasets used and/or analyzed during the current study are available on reasonable request and according to procedures as stipulated on www.giantt.nl. Requests to access these datasets could be directed to p.denig@umcg.nl.
