# Supplementary material for: Older Age, Polypharmacy, and Low Systolic Blood Pressure Are Associated With More Hypotension-Related Adverse Events in Patients With Type 2 Diabetes Treated With Antihypertensives
Source: Front Pharmacol. 2021 Sep 24;12:728911. doi: 10.3389/fphar.2021.728911 (PMC8497792; doi:10.3389/fphar.2021.728911)
Supplement: Supplementary file 1 [file DataSheet1.docx]

Supplementary Material

# Flow chart of included patients


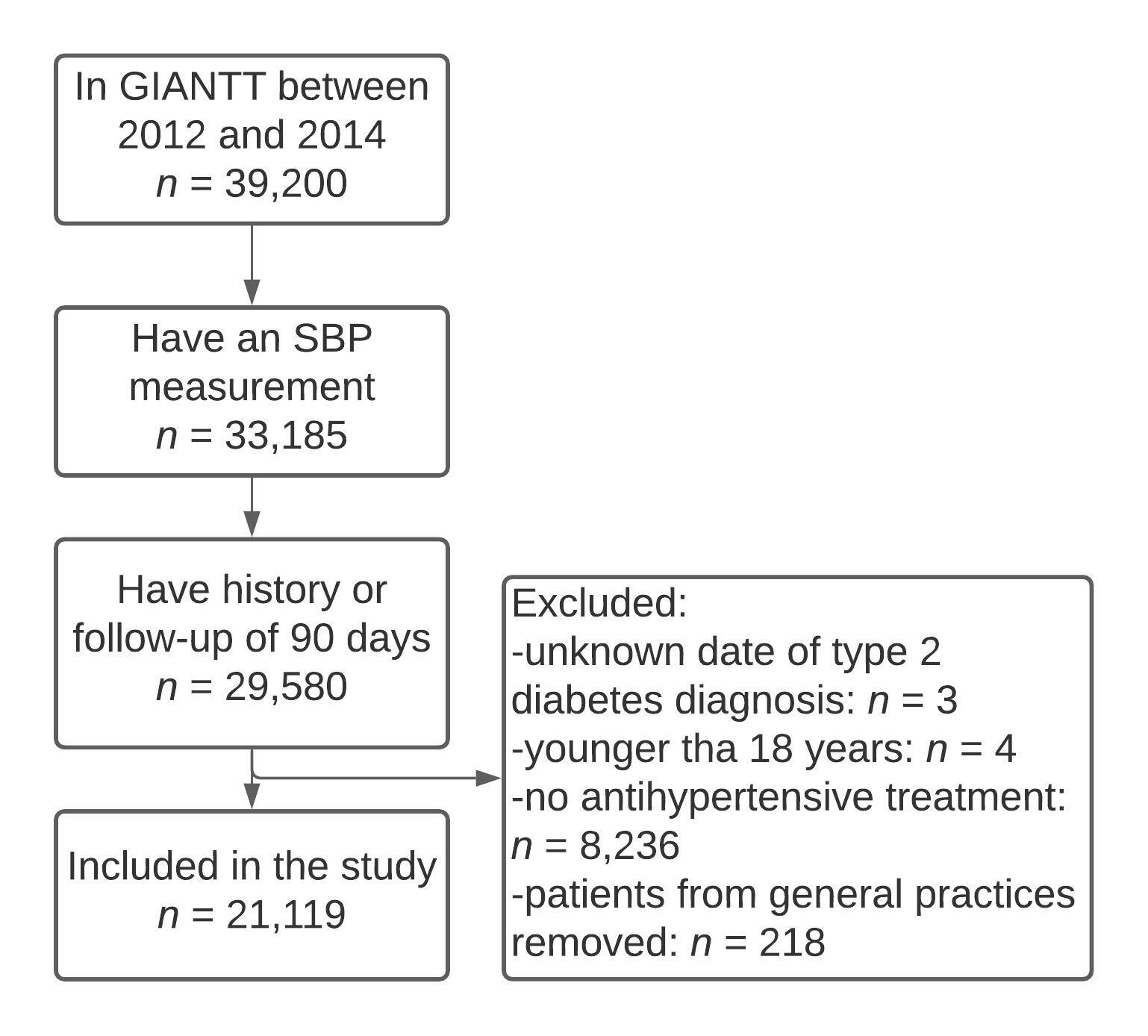


**FigS1:** Included patients based on the inclusion criteria. GIANTT = Groningen Initiative to ANalyse Type 2 diabetes Treatment; SBP = systolic blood pressure.

# Sensitivity analyses using different cut-off values for age

## Cut-off 80 years


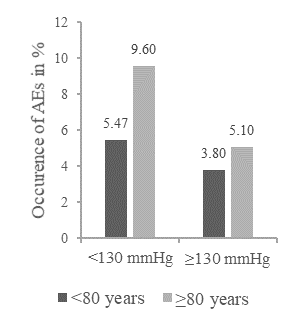


| SBP at index date (mmHg)* | Age (years) | |
| --- | --- | --- |
|  | <80 | ≥80 |
| <130 | 576/10528 | 229/2353 |
| ≥130 | 255/6709 | 78/1529 |
| AEs (%) | 4.8 | 7.8 |

**FigS2:** Occurrence of hypotension-related adverse events (AEs) per systolic blood pressure (SBP) level for patients aged <80 years and ≥80 years. The table below presents the numbers of AEs per total number of patients in that group. *Index date is defined as the lowest SBP between 2012 and 2014.


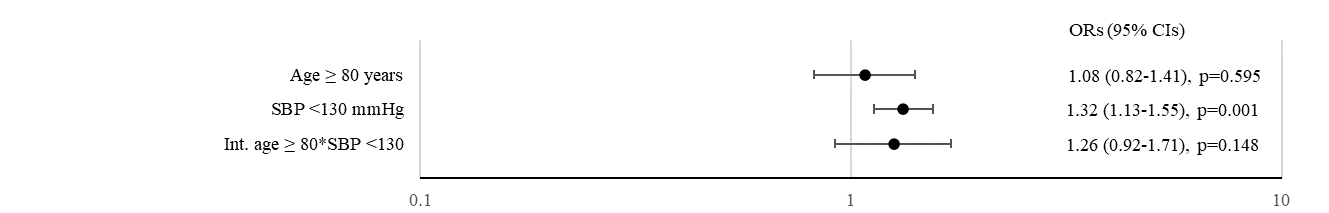


**FigS3:** Odds ratios (OR) with 95% confidence intervals (CIs) and p-values for age, systolic blood pressure (SBP) and their interaction. This analysis was adjusted for glycated hemoglobin, diabetes duration, body mass index, smoking, diastolic blood pressure, estimated glomerular filtration rate, glucose lowering therapy, dyslipidemia, lipid lowering therapy, albuminuria, number of comedication and sex. Int.=interaction.

# Sensitivity analyses using different cut-off values for systolic blood pressure (SBP)

## Cut-off 120 mmHg


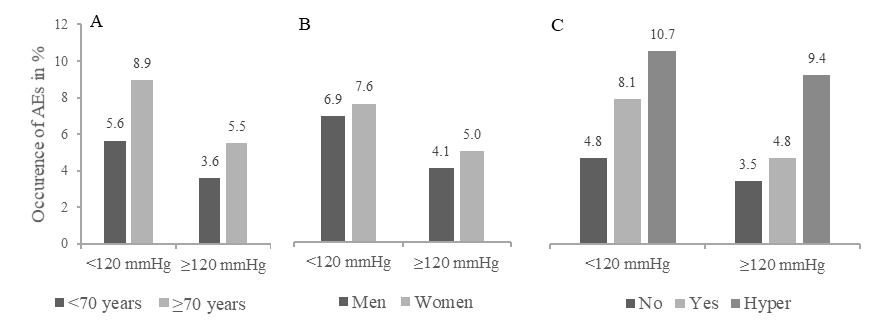


| SBP at index date (mmHg)* | Age (years) | | Sex | | Polypharmacy | | |  |
| --- | --- | --- | --- | --- | --- | --- | --- | --- |
|  | <70 | ≥70 | Men | Women | No | Yes | Hyper | Total AEs (%) |
| <120 | 185/3279 | 284/3177 | 211/3057 | 258/3399 | 111/2328 | 255/3166 | 103/962 | 7.3 |
| ≥120 | 265/7402 | 401/7261 | 292/7155 | 374/7508 | 240/6841 | 319/6685 | 107/1137 | 4.5 |

**FigS4:** Occurrence of hypotension-related adverse events (AEs) per systolic blood pressure (SBP) level by (A) age, (B) sex and (C) polypharmacy. The table below presents the numbers of AEs per total number of patients in that group. *Index date is defined as the lowest SBP level between 2012 and 2014.

^
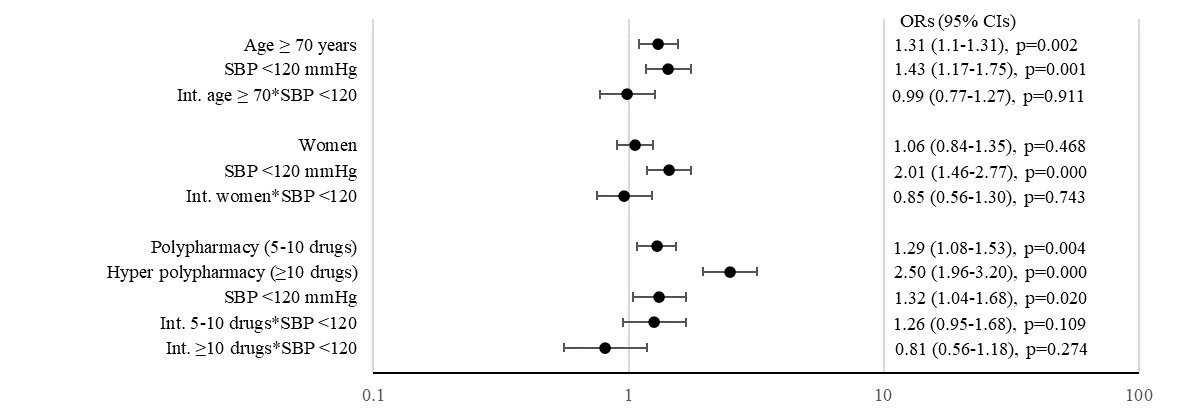
^

**FigS5:** Odds ratios (OR) with 95% confidence intervals (CIs) and p-values for age, sex, polypharmacy, and systolic blood pressure (SBP) and interactions. Age and sex analyses were adjusted for glycated hemoglobin, diabetes duration, body mass index, smoking, diastolic blood pressure, estimated glomerular filtration rate, glucose lowering therapy, dyslipidemia, lipid lowering therapy, albuminuria, number of comedication and sex or age; polypharmacy analysis was adjusted for the same variables except for glucose and lipid lowering therapy. Int.=interaction

## Cut-off 140 mmHg


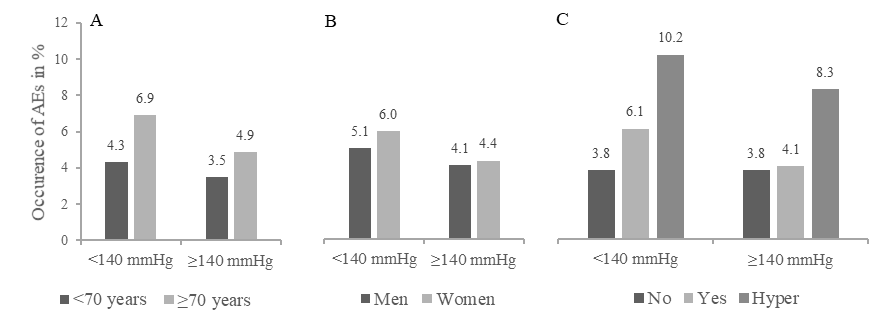


| SBP at index date (mmHg)* | Age (years) | | Sex | | Polypharmacy | | |  |
| --- | --- | --- | --- | --- | --- | --- | --- | --- |
|  | <70 | ≥70 | Men | Women | No | Yes | Hyper | Total AEs (%) |
| <140 | 402/9309 | 598/8644 | 440/8689 | 560/9264 | 293/7649 | 517/7928 | 190/1859 | 5.6 |
| ≥140 | 48/1372 | 87/1794 | 63/1523 | 72/1643 | 58/1520 | 57/1406 | 20/240 | 4.3 |

**FigS6:** Occurrence of hypotension-related adverse events (AEs) per systolic blood pressure (SBP) level by (A) age, (B) sex and (C) polypharmacy. The table below presents the numbers of AEs per total number of patients in that group. *Index date is defined as the lowest SBP level between 2012 and 2014.


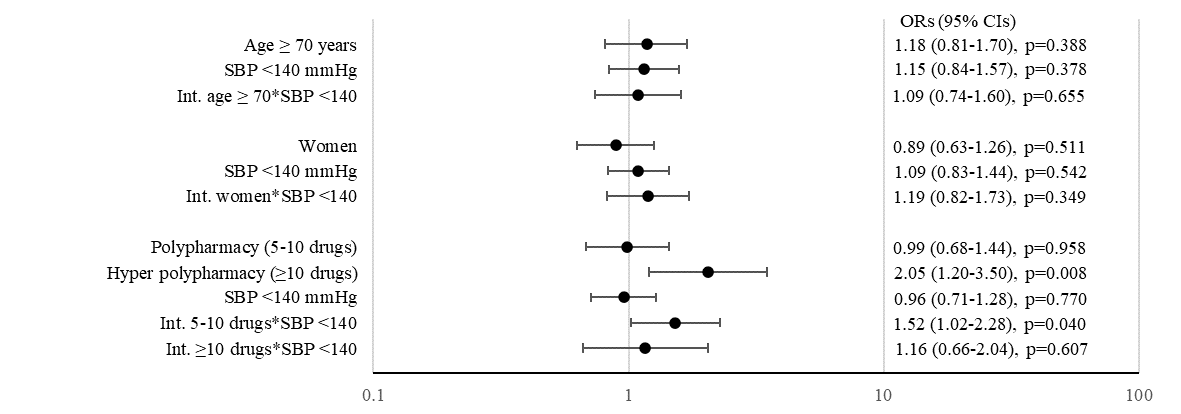


**FigS7:** Odds ratios (OR) with 95% confidence intervals (CIs) and p-values for age, sex, polypharmacy, and systolic blood pressure (SBP) and interactions. Age and sex analyses were adjusted for glycated hemoglobin, diabetes duration, body mass index, smoking, diastolic blood pressure, estimated glomerular filtration rate, glucose lowering therapy, dyslipidemia, lipid lowering therapy, albuminuria, number of comedication and sex or age; polypharmacy analysis was adjusted for the same variables except for glucose and lipid lowering therapy. Int.=interaction

# Sensitivity analysis using an extended list of adverse events

**Table S1:** Presence of additional adverse event; N (%); N = 1,588

| Postural hypotension (K88) | 534 (34) |  |
| --- | --- | --- |
| Weakness, tiredness (A04) | 336 (21) |  |
| Dizziness, vertigo (N17) | 229 (14) |  |
| Musculoskeletal injury (L81) | 166 (10) |  |
| Syncope (A06) | 117 (7) |  |
| Abrasion, scratch (S17) | 110 (7) |  |
| Trauma, injury (A80) | 109 (7) |  |
| Other fracture (L76) | 87 (5) |  |
| Bruises, concussion (S16) | 74 (5) |  |
| Femur fracture (L75) | 27 (2) |  |


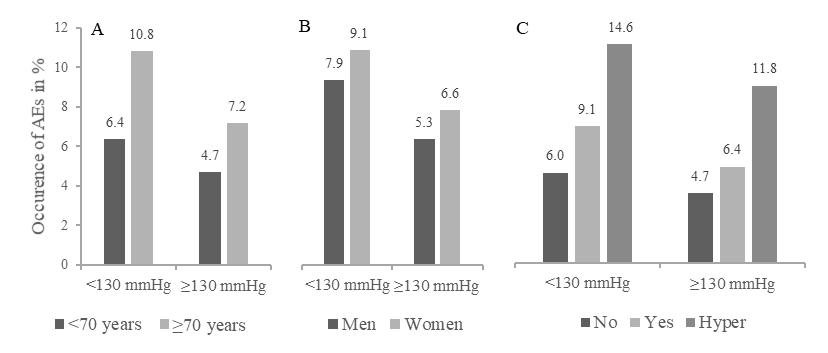


| SBP at index date (mmHg)* | Age (years) | | Sex | | Polypharmacy | | |  |
| --- | --- | --- | --- | --- | --- | --- | --- | --- |
|  | <70 | ≥70 | Men | Women | No | Minor | Major | Total AEs (%) |
| <130 | 426/6678 | 670/6203 | 485/6177 | 611/6704 | 316/5232 | 560/6141 | 220/1508 | 8.5 |
| ≥130 | 188/4003 | 304/4235 | 215/4035 | 277/4203 | 183/3937 | 239/3710 | 70/591 | 6.0 |
| Total AEs (%) | 5.8 | 9.3 | 6.9 | 8.1 | 5.4 | 8.1 | 13.8 | 7.5 |

**FigS8:** Occurrence of hypotension-related adverse events (AEs) per systolic blood pressure (SBP) level by (A) age, (B) sex and (C) polypharmacy. The table below presents the numbers of AEs per total number of patients in that group. *Index date is defined as the lowest SBP level between 2012 and 2014.


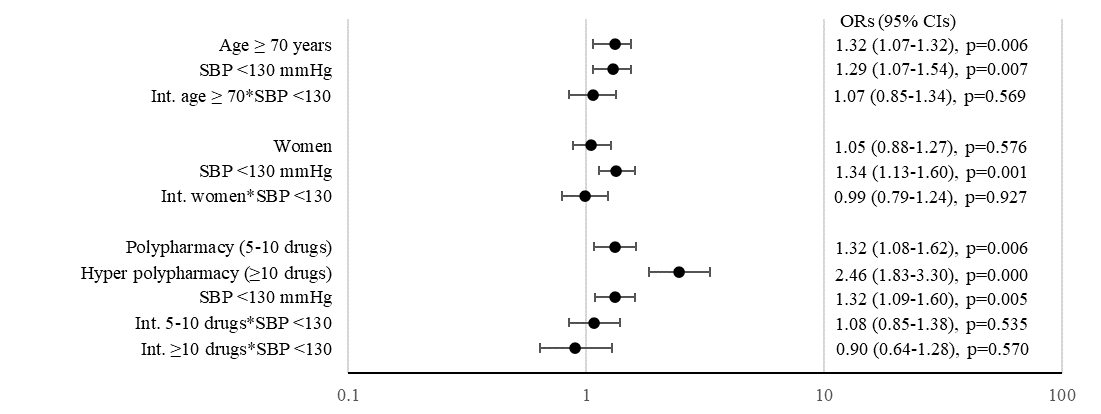


**FigS9:** Odds ratios (OR) with 95% confidence intervals (CIs) and p-values for age, sex, polypharmacy, and systolic blood pressure (SBP) and interactions. Age and sex analyses were adjusted for glycated hemoglobin, diabetes duration, body mass index, smoking, diastolic blood pressure, estimated glomerular filtration rate, glucose lowering therapy, dyslipidemia, lipid lowering therapy, albuminuria, number of comedication and sex or age; polypharmacy analysis was adjusted for the same variables except for glucose and lipid lowering therapy. Int.=interaction

# Post hoc analysis using only adverse events which occurred in the 90 days after or at the index date


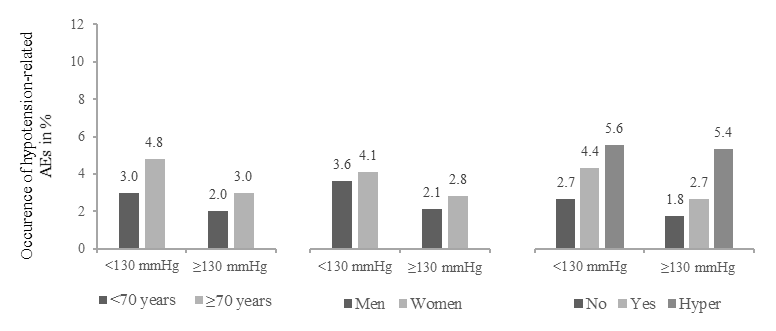


| SBP at index date (mmHg)* | Age (years) | | Sex | | Polypharmacy | | |  |
| --- | --- | --- | --- | --- | --- | --- | --- | --- |
|  | <70 | ≥70 | Men | Women | No | Minor | Major | Total AEs (%) |
| <130 | 203/6678 | 295/6203 | 224/6177 | 274/6704 | 143/5232 | 270/6141 | 85/1508 | 3.9 |
| ≥130 | 79/4003 | 126/4235 | 86/4035 | 119/4203 | 72/3937 | 101/3710 | 32/591 | 2.5 |
| Total AEs (%) | 2.6 | 4.0 | 3.0 | 3.6 | 2.3 | 3.8 | 5.6 | 3.3 |

**FigS10:** Occurrence of hypotension-related adverse events (AEs) which occurred at or after the index date per systolic blood pressure (SBP) level by (A) age, (B) sex and (C) polypharmacy. The table below presents the numbers of AEs per total number of patients in that group. *Index date is defined as the lowest SBP level between 2012 and 2014.


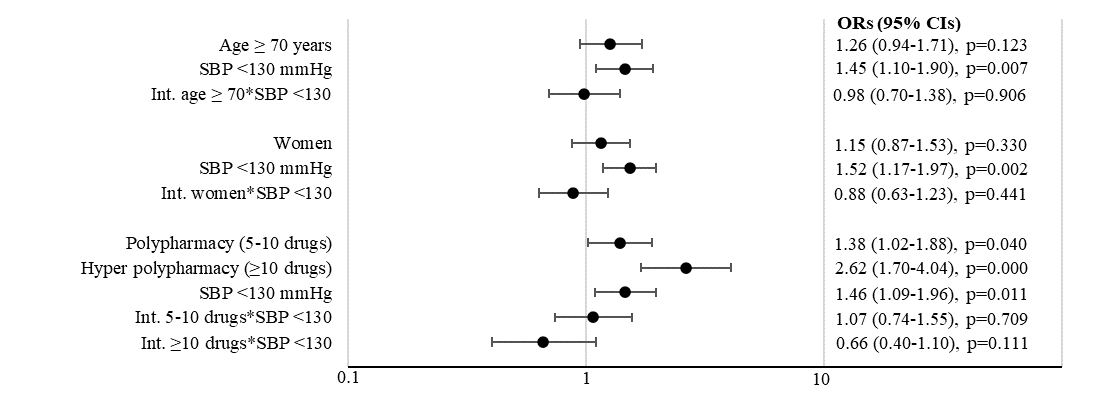


**FigS11:** Odds ratios (OR) with 95% confidence intervals (CIs) and p-values for age, sex, polypharmacy, and systolic blood pressure (SBP) and interactions. Age and sex analyses were adjusted for glycated hemoglobin, diabetes duration, body mass index, smoking, diastolic blood pressure, estimated glomerular filtration rate, glucose lowering therapy, dyslipidemia, lipid lowering therapy, albuminuria, number of comedication and sex or age; polypharmacy analysis was adjusted for the same variables except for glucose and lipid lowering therapy. Int.=interaction
